# Circulating angiopoietin-like protein 8 (ANGPTL8) and ANGPTL3 concentrations in relation to anthropometric and metabolic profiles in Korean children: a prospective cohort study

**DOI:** 10.1186/s12933-015-0324-y

**Published:** 2016-01-06

**Authors:** Hye Soo Chung, Min Jung Lee, Soon Young Hwang, Hyun Jung Lee, Hye Jin Yoo, Ji-A Seo, Sin Gon Kim, Nan Hee Kim, Sei Hyun Baik, Dong Seop Choi, Seon Mi Kim, Kyung Mook Choi

**Affiliations:** Division of Endocrinology and Metabolism, Department of Internal Medicine, College of Medicine, Korea University, Seoul, Korea; Department of Biostatistics, College of Medicine, Korea University, Seoul, Korea; Department of Family Medicine, College of Medicine, Korea University, Seoul, Korea

**Keywords:** ANGPTL3, ANGPTL8, Cholesterol, Dyslipidemias, HDL, LDL, Triglycerides

## Abstract

**Background:**

Previous studies have shown that angiopoietin-like protein 8 (ANGPTL8), also called as betatrophin, acts together with ANGPTL3 to regulate lipid metabolism, glucose metabolism, and energy homeostasis. Moreover, ANGPTL8 promotes proliferation of pancreatic β-cells and induces insulin secretion. However, there are no previous longitudinal studies in humans.

**Methods:**

We analyzed the age- and sex-matched data of 240 normal weight and overweight Korean children from the Korean Metabolic disorders and Obesity Study in Elementary School children (K-MOSES), a prospective observational cohort study.

**Results:**

At baseline, ANGPTL8 concentrations were positively associated with triglycerides (TG) (*r* = 0.168, *P* = 0.010), whereas ANGPTL3 levels were associated with fasting insulin (*r* = 0.248, *P* < 0.001) and the homeostasis model assessment of insulin resistance (HOMA-IR) (*r* = 0.197, *P* = 0.002). Although both ANGPTL8 and ANGPTL3 levels did not differ between children with normal weight and children with overweight, ANGPTL8 levels were increased in males compared to females (341.2 [267.4–436.5] vs. 270.2 [213.9–378.8] pg/ml, *P* = 0.001). In particular, there was no significant inter-relationship between circulating ANGPTL8 and ANGPTL3 concentrations in Korean boys and girls (*r* = −0.073, *P* = 0.265). Multivariate analysis showed that baseline ANGPTL8 concentrations were independently associated with future changes of serum TG levels in Korean children after adjusting for confounding factors after a 3 year follow-up period (*r* = −0.165, *P* = 0.016).

**Conclusions:**

This longitudinal study demonstrated for the first time that baseline ANGPTL8 levels were associated with baseline and future changes in TG levels in Korean children.

## Background

Recently, angiopoietin-like protein 8 (ANGPTL8) was identified as a novel hormone that induces a remarkable dose-dependent increase in pancreatic β-cell proliferation [1]. Also known as betatrophin, lipasin, and refeeding-induced fat and liver protein (RIFL), other researchers have also reported the identification and function of ANGPTL8 [2]. ANGPTL8, which is expressed in liver and adipose tissue, is upregulated by feeding and suppressed by fasting in mice and humans [3, 4]. Also, in one study, adenovirus-induced overexpression of ANGPTL8 resulted in increased serum triglyceride (TG) levels in mice [3]. Ren et al. reported the induction of the ANGPTL8 gene during adipogenesis and knockdown of the ANGPTL8 gene led to reduced adipogenesis and reduced TG storage [4]. In addition, there is evidence that *ob/ob* mice have a significant increase in ANGPTL8 transcript levels in their adipose tissue [4]. Importantly, a recent cross-sectional study demonstrated that ANGPTL8 concentrations were increased in the plasma of patients with type 1 diabetes compared to controls [5]. Furthermore, Hu et al. reported that serum ANGPTL8 levels were significantly higher in patients with type 2 diabetes than in healthy control subjects [6]. However, Gusarova et al. showed that overexpression of ANGPTL8 in livers of mice did not change β-cell expansion or glucose metabolism, although it doubled plasma TG levels [7]. Therefore, further research is needed for evaluating the role of ANGPTL8 in human metabolic disorders.

ANGPTL3 is an important regulator of lipoprotein metabolism, and deletion or overexpression of ANGPTL8 and ANGPTL3 have similar effects on lipid profiles in mice [8]. Quagliarini et al. demonstrated that ANGPTL8 interacts with ANGPTL3 and facilitates its cleavage [9]; ANGPTL3 needs to be cleaved to release the functional N-termini, which inhibits lipoprotein lipase (LPL) and results in reduced TG clearance and a phenotype with greater TG stores [10]. Although plasma TG levels did not change in mice overexpressing ANGPTL3 alone, co-overexpression of ANGPTL3 and ANGPTL8 resulted in hypertriglyceridemia. Furthermore, overexpression of ANGPTL8 in ANGPTL3-knockout mice failed to promote hypertriglyceridemia [9]. These results indicate that there is a close interaction between ANGPTL3 and ANGPTL8 in mice, but there is no previous report about their inter-relationship in humans. Furthermore, betatrophin has been mainly investigated in older adults, and there are no studies on the children. To the best of our knowledge, there is no longitudinal study that examines the association between ANGPTLs and changes in metabolic profiles. In the present study, we analyzed data from a prospective observational study to examine the impact of ANGPTL3 and ANGPTL8 in relation to baseline and follow-up anthropometric and metabolic profiles in Korean boys and girls.

## Methods

### Study subjects

Study subjects were selected from the Korean Metabolic disorders and Obesity Study in Elementary School children (K-MOSES), an observational cohort study to examine children annually since 2006 at eight elementary schools in Seoul, South Korea. The main aim of K-MOSES is to comprehensively and prospectively assess obesity-related metabolic risk factors and to evaluate clinical outcomes in Korean children. The details of the cohort were described previously [11, 12]. We recruited 457 children, aged 9 year at baseline, who participated in a school-based health examination in 2007 and reexamined at a 3 year follow-up assessment in 2010. Subjects with insufficient anthropometric and biochemical laboratory data (n = 137) were excluded, so data from 320 subjects (164 males and 156 females) were analyzed in the present study. A total of 52 (14 males and 38 females) out of 320 subjects (16.3 %) were defined as overweight. As we conducted a case–control analysis matched for age and sex, 28 overweight subjects (14 males and 14 females) were randomly selected as cases and 212 normal weight subjects were enrolled as controls (106 males and 106 females). None of the children had a history of cardiovascular disease, diabetes, hypertension, or endocrine disorders, and they were nonsmokers. Written informed consent was obtained from their parents and the Korea University Institutional Review Board approved this study protocol in accordance with the Declaration of Helsinki of the World Medical Association (approval No. KUGH 11004-001).

### Anthropometric and laboratory measurements

During both visits anthropometric measurements were obtained from all children in light clothing without shoes. Height and weight were measured by an automatic height-weight scale, to the nearest 0.5 cm and 0.5 kg, respectively; BMI was computed as weight (kilograms)/height (meters). Waist circumference was measured at the midpoint between the lower border of the rib cage and the top of the lateral border of the iliac crest. We defined normal weight (girl’s BMI < 19.88, boy’s BMI < 20.76) among children on the basis of the Korean Pediatric Society 2005 guidelines, which defines as having a BMI below the 85th percentile for age and sex [13]. Blood pressure was measured with a standard brachial cuff technique. Self-reported information on pubertal development was collected, using a Tanner drawing of the five stages of pubertal development, which involved the development of genitalia and pubic hair [14]. All blood samples were obtained the morning after an 8 h overnight fast, and were immediately stored at −80 °C for subsequent analysis. Serum total cholesterol, TG, and high-density lipoprotein cholesterol (HDL-C) levels were determined enzymatically using a model 747 chemistry analyzer (Hitachi, Tokyo, Japan). AST and ALT levels were determined enzymatically with the aid of an auto-analyzer (TBA-200FR; Toshiba, Tokyo, Japan). The glucose oxidase method was used to measure plasma glucose levels. The homeostasis model assessment of insulin resistance (HOMA-IR) was calculated as [fasting insulin (in microunits per milliliter) × fasting glucose (in mmol per liter)]/22.5. Serum ANGPTL3 concentrations were measured with an enzyme-linked immunosorbent assay (R&D systems, Minneapolis, MN, USA), and the intra- and inter-assay CVs were 2.7–4.1 % and 6.7–8.5 %, respectively. Serum ANGPTL8 concentrations were measured with an enzyme-linked immunosorbent assay (EIAAB, Wuhan, China) and the intra- and inter-assay CVs were less than 4.8 and 7.2 %, respectively.

### Statistical analysis

Continuous variables with normal distributions are expressed as the mean ± SD, whereas continuous variables with skewed distributions are expressed as the median and interquartile range. Baseline demographic and biochemical characteristics of the study population by sex were compared using an independent *t* test or the Mann–Whitney U test for continuous variables. Because serum levels of ANGPTL3 and ANGPTL8 were not normally distributed, log transformed values were used for the analysis. To evaluate the associations between hepatokines and anthropometric and metabolic variables at baseline, Pearson’s correlations were used. Unadjusted Pearson’s correlations and Pearson’s partial correlations adjusted for confounding factors were used to evaluate the association between ANGPTLs and changes in metabolic variables at the 3 year follow up. All statistical analyses were performed using SPSS version 20.0 for Windows (SPSS Inc., Chicago, IL, USA). A *P* value of <0.05 was considered statistically significant.

## Results

### Characteristics of study subjects

**Fig. 1:**
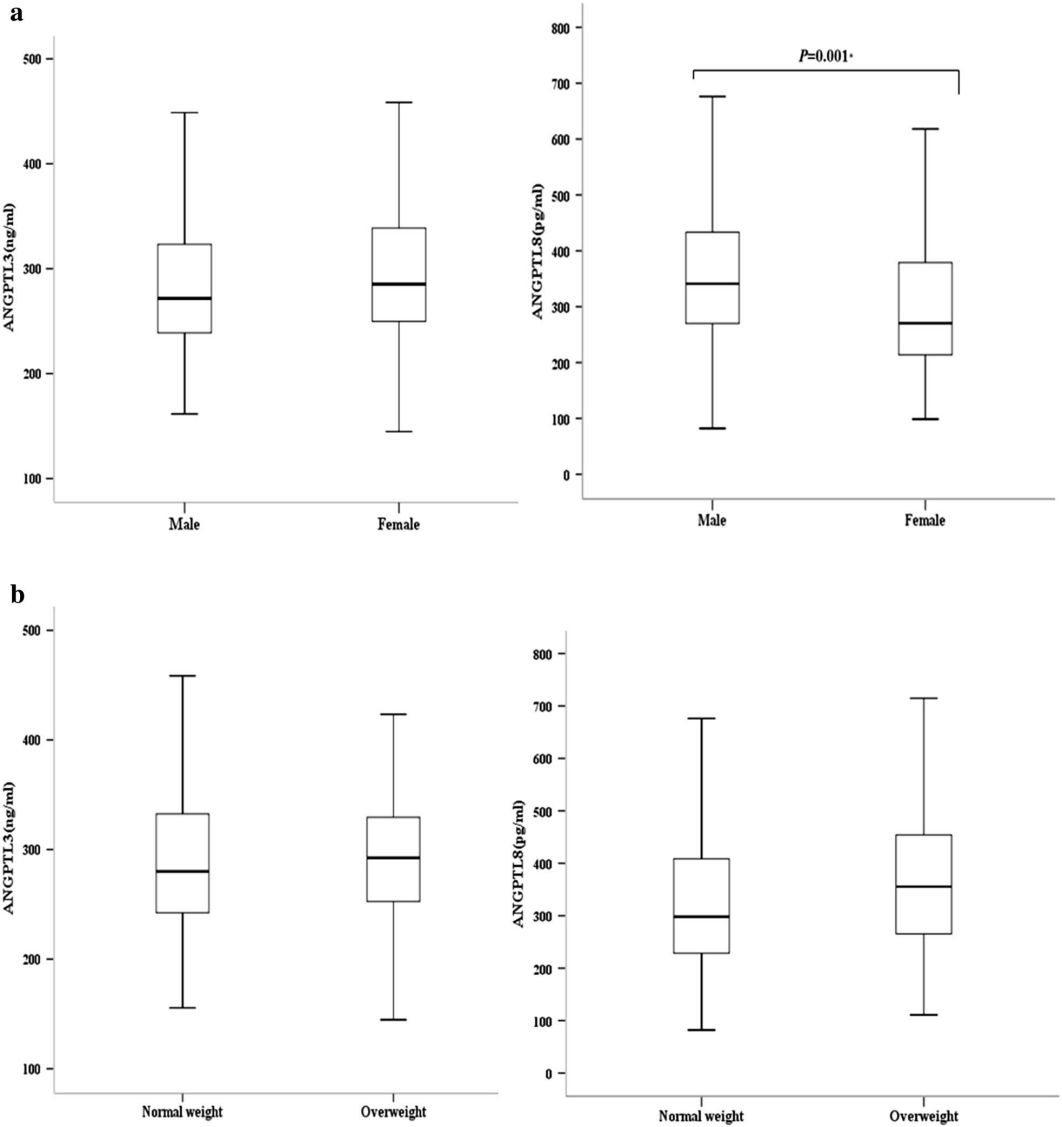
Comparison of circulating ANGPTL concentrations under different baseline conditions. **a** Male (n = 120) vs. female (n = 120); ANGPTL3 (271.7 [238.9–323.2] vs. 285.2 [249.8–338.8] ng/ml, *P* = 0.117), and ANGPTL8 (341.2 [267.4–436.5] vs. 270.2 [213.9–378.8] pg/ml, *P* = 0.001). **b** Normal weight (n = 212) vs. overweight (n = 28); ANGPTL3 (280.0 [242.4–332.5] vs. 292.4 [252.7–329.5] ng/ml, *P* = 0.500), and ANGPTL8 (298.0 [228.8–408.7] vs. 355.4 [265.3–473.1] pg/ml, *P* = 0.283, *P < 0.05

Anthropometric and metabolic characteristics of the study participants at baseline and 3 year later are presented in Table 1. At baseline, girls were taller and heavier than boys, and BMI and waist circumference were also greater in girls. However, blood pressure and laboratory measurements were similar except for fasting insulin levels; girls had significantly higher insulin levels than boys. With that said, HOMA-IR and HOMA-β values were not different between boys and girls. In particular, ANGPTL8 concentrations were significantly greater in boys compared to girls, whereas ANGPTL3 levels were not different (Fig. 1a). On the other hand, we did find any significant differences in either ANGPTL8 or ANGPTL3 levels between children with normal weight and overweight children (Fig. 1b).

**Table 1:** Anthropometric and metabolic characteristics of study participants at baseline and after 3 year

	Baseline			3 year later		
	Male (n = 120)	Female (n = 120)	*P*	Male (n = 120)	Female (n = 120)	*P*
Height (cm)	130.6 (128.8−131.9)	136.0 (133.8−138.6)	<0.001^b^	150.8 ± 7.0	153.1 ± 5.3	0.004^a^
Weight (kg)	28.5 (26.1−31.3)	31.9 (28.2−36.5)	<0.001^b^	43.4 (36.6−51.2)	43.4 (40.8−49.3)	0.213^b^
BMI (kg/m^2^)	16.9 (15.4−18.4)	17.5 (16.2−19.2)	0.047^b^	19.2 (17.4−22.0)	19.6 (17.9−21.9)	0.298^b^
WC (cm)	57.0 (53.8−61.0)	59.5 (56.0−64.0)	0.004^b^	65.0 (59.6−74.5)	66.0 (61.7−72.9)	0.625^b^
SBP (mmHg)	105.5 (98.0−110.0)	105.0 (100.0−112.0)	0.676^b^	110.0 (110.0−120.0)	115.0 (110.0−120.0)	0.032^b^
DBP (mmHg)	66.0 (60.0−70.0)	65.5 (60.0−72.0)	0.544^b^	70.0 (65.0−80.0)	75.0 (70.0−80.0)	0.022^b^
FPG (mmol/L)	4.6 (4.3−4.9)	4.7 (4.5−5.0)	0.376^b^	4.1 ± 0.4	4.1 ± 0.3	0.584^a^
Insulin (μIU/ml)	6.6 (5.0−8.0)	7.4 (5.7−9.1)	0.026^b^	–	–	–
HOMA-IR	1.4 (1.0−1.7)	1.5 (1.1−2.0)	0.110^b^	–	–	–
HOMA-β	110.3 (80.6−158.4)	115.3 (79.9−165.2)	0.893^b^	–	–	–
TC (mmol/L)	4.3 (3.9−4.7)	4.3 (3.8−4.7)	0.527^b^	3.9 (3.5−4.4)	4.0 (3.7−4.4)	0114^b^
TG (mmol/L)	0.7 (0.5−1.0)	0.7 (0.5−1.0)	0.324^b^	0.8 (0.6−1.1)	0.9 (0.8−1.2)	<0.001^b^
HDL-C (mmol/L)	1.5 (1.2−1.7)	1.5 (1.3−1.6)	0.942^b^	1.4 (1.2−1.7)	1.4 (1.2−1.5)	0.133^b^
AST (IU/L)	26.0 (23.0−30.0)	26.0 (22.0−29.0)	0.326^b^	25.0 (23.0−29.)	21.0 (18.0−24.0)	<0.001^b^
ALT (IU/L)	22.0 (20.0−25.0)	23.0 (21.0−26.0)	0.406^b^	14.0 (12.0−18.0)	11.0 (10.0−13.0)	<0.001^b^

All values are mean ± standard deviation or median and interquartile range

*BMI* body mass index, *WC* waist circumference, *SBP* systolic blood pressure, *DBP* diastolic blood pressure, *FPG* fasting plasma glucose, *TC* total cholesterol, *TG* triglycerides, *HDL-C* high-density lipoprotein cholesterol, *AST* aspartate aminotransferase, *ALT* alanine aminotransferase

^a^ Independent t-test or ^b^ the Mann–Whitney U

### Correlation analysis between ANGPTLs and clinical variables at baseline

Table 2 shows Pearson’s correlation analysis between log-transformed ANGPTL concentrations and clinical variables at baseline. ANGPTL3 levels were positively correlated with fasting insulin (*r* = 0.248, *P* < 0.001) and HOMA-IR (*r* = 0.197, *P* = 0.002) values as well as ALT levels (*r* = 0.131, *P* = 0.042). However, anthropometric measurements and laboratory values including glucose and lipid profiles were not correlated with ANGPTL3 concentrations. In contrast, ANGPTL8 concentrations showed a positive relationship with triglyceride levels (*r* = 0.168, *P* = 0.010). However, other anthropometric and laboratory measurements were not correlated with ANGPTL8 levels. In particular, ANGPTL8 levels were not associated with HOMA-β (*r* = 0.006, *P* = 0.927) values, which reflect insulin secretory capacity. Interestingly, there was no significant inter-relationship between circulating ANGPTL8 and ANGPTL3 concentrations in Korean children (*r* = −0.073, *P* = 0.265).

**Table 2:** Pearson’s correlations between log-transformed ANGPTLs and anthropometric and metabolic variables at baseline

	ANGPTL3		ANGPTL8	
	*r*	*P*	*r*	*P*
Height (cm)	−0.059	0.366	−0.111	0.089
Weight (kg)	−0.038	0.562	−0.068	0.302
BMI (kg/m^2^)	−0.016	0.804	−0.017	0.789
WC (cm)	−0.002	0.978	−0.071	0.278
SBP (mmHg)	0.023	0.723	0.111	0.090
DBP (mmHg)	0.094	0.145	0.009	0.886
FPG (mmol/L)	−0.112	0.084	0.093	0.153
TC (mmol/L)	−0.045	0.490	0.035	0.595
TG (mmol/L)	0.042	0.521	0.168	0.010*
HDL-C (mmol/L)	−0.016	0.804	−0.053	0.414
AST (IU/L)	0.108	0.094	0.091	0.162
ALT (IU/L)	0.131	0.042*	−0.022	0.732
Insulin (μIU/ml)	0.248	<0.001*	−0.010	0.881
HOMA-IR	0.197	0.002*	0.018	0.784
HOMA-β	0.055	0.401	0.006	0.927

*BMI* body mass index, *WC* waist circumference, *SBP* systolic blood pressure, *DBP* diastolic blood pressure, *FPG* fasting plasma glucose, *TC* total cholesterol, *TG* triglycerides, *HDL-C* high-density lipoprotein cholesterol, *AST* aspartate aminotransferase, *ALT* alanine aminotransferase, *HOMA-IR* homeostasis model assessment of insulin resistance

* *P* < 0.05

### Multivariate analysis for the associations between baseline ANGPTLs and changes in clinical variables after a 3 year follow-up period

Next, we analyzed the association between baseline ANGPTL concentrations and changes in anthropometric and laboratory measurements at the end of the 3 year follow-up period (Table 3). In an unadjusted model, baseline ANGPTL8 concentrations were significantly associated with future changes in triglyceride levels (*r* = −0.166, *P* = 0.011), although other laboratory and anthropometric values were not correlated with ANGPTL8 levels. This relationship persisted after adjustment for sex and Tanner stage (*r* = −0.191, *P* = 0.005). Furthermore, the statistical significance was maintained after further adjustment for BMI, SBP, and FPG (*r* = −0.165, *P* = 0.016). However, ANGPTL3 concentrations were not associated with any changes in clinical variables in both the unadjusted and adjusted models.

**Table 3:** Pearson’s correlations for the association between baseline ANGPTL concentrations and changes (Δ) in anthropometric and metabolic variables after a 3 year follow-up period

	Unadjusted model				Adjusted for sex, tanner stage, BMI, SBP, and FPG			
	ANGPTL3		ANGPTL8		ANGPTL3		ANGPTL8	
	*r*	*P*	*r*	*P*	*r*	*P*	*r*	*P*
ΔHeight	0.124	0.055	0.045	0.488	0.153	0.025*	−0.028	0.688
ΔWeight	0.104	0.110	0.032	0.625	0.104	0.128	−0.013	0.850
ΔBMI	−0.007	0.915	0.081	0.212	−0.020	0.768	0.072	0.292
ΔWC	−0.010	0.878	−0.009	0.893	−0.042	0.542	0.015	0.827
ΔSBP	0.002	0.973	−0.093	0.157	0.016	0.816	−0.032	0.640
ΔDBP	−0.034	0.604	−0.074	0.258	−0.040	0.564	−0.033	0.631
ΔFPG	0.125	0.053	−0.075	0.251	0.020	0.771	−0.035	0.606
ΔTC	0.083	0.203	−0.039	0.551	0.071	0.304	0.028	0.685
ΔTG	0.098	0.131	−0.166	0.011*	0.066	0.335	−0.165	0.016*
ΔHDL-C	−0.101	0.119	0.011	0.867	−0.102	0.137	0.015	0.823

*BMI* body mass index, *WC* waist circumference, *SBP* systolic blood pressure, *DBP* diastolic blood pressure, *FPG* fasting plasma glucose, *TC* total cholesterol, *TG* triglycerides, *HDL-C* high-density lipoprotein cholesterol

* *P* < 0.05

## Discussion

In the present study, we found that baseline ANGPTL8 levels were associated with baseline and future changes in TG levels in Korean children. However, there was no significant inter-relationship between circulating ANGPTL8 and ANGPTL3 concentrations.

Angiopoietin-like proteins (ANGPTLs) are a family of proteins structurally similar to the angiopoietins and have important roles in lipid and glucose metabolism, inflammation, atherosclerosis, and hematopoiesis [8, 15, 16]. Eight members have been identified so far and denominated as ANGPTLs 1–8 [8]. Among ANGPTLs, ANGPTL2 can be considered a key mediator that links obesity and atherosclerosis [8, 16]. Particularly ANGPTL3, ANGPTL8 and ANGPTL4 studied for a major role in TG regulator and in insulin mediator [8, 17].

ANGPTL8 has been suggested as a potential target for β-cell regenerative therapy in diabetes. However, contradictory results of ANGPTL8 in glucose metabolism have been reported in patients with type 2 diabetes. Some studies found that serum ANGPTL8 levels were elevated in patients with type 2 diabetes compared to subjects with nondiabetic subjects [6, 18–20]. Furthermore, Chen et al. observed that ANGPTL8 levels were positively correlated with insulin resistance indexes including HOMA-IR [18]. Hu et al. showed that age, 2 h post-OGTT glucose and postprandial serum insulin were independent factors that were associated with serum ANGPTL8 levels. Ebert et al. demonstrated that treatment of 3T3-L1 adipocytes with insulin induced ANGPTL8 expression in a dose-dependent manner [19]. Gao et al. reported that ANGPTL8 are increased in newly diagnosed T2DM patients and decreased in patients with comparatively better islet beta cell function [21]. However, other studies reported decreased levels of betatrophin in T2DM through insulin resistance [22, 23]. Gokulakrishnan et al. reported that ANGPTL8/betatrophin levels are lower in youth-onset type 2 diabetes mellitus [22]. By contrast, Fenzl et al. reported that ANGPTL8 levels are similar when comparing lean and morbidly obese patients or when comparing non-diabetic and type 2 diabetic participants [24]. Although the exact reason for this discrepancy has not been clearly defined, a recent study suggested that circulating ANGPTL8 concentrations do not mirror insulin action in either the liver or adipose tissue [17]. In the present study, we did not find a significant relationship between baseline ANGPTL8 levels and future changes in fasting glucose and obesity parameters such as BMI and waist circumference. Furthermore, ANGPTL8 concentrations were not associated with HOMA-β or HOMA-IR values and did not differ between normal weight and overweight children. Most previous ANGPTL human studies have been investigated in older adults, there is limited data of ANGPTL levels in children. Studies in younger age participants may be valuable, because they may be less influenced by various comorbid status and lifestyle factors.

Previous studies have shown that ANGPTL8 regulates lipid metabolism. Fenzl et al. found that ANGPTL8 concentrations are correlated with atherogenic lipid profiles [24]. Gusarova et al. reported that plasma TG levels have been shown to be reduced in response to ANGPTL8 deletion and greater in response to overexpression of ANGPTL8 [7]. Collectively, these results suggest that inhibition of ANGPTL8 could be a therapeutic strategy to ameliorate hypertriglyceridemia. Wang et al. also reported that mice lacking ANGPTL8 exhibited disrupted TG metabolism [25]. However, there has been controversy in terms of if and how ANGPTL8 regulates lipid metabolism. Ren et al. demonstrated that ANGPTL8 is lipogenic [4]. Quagliarini et al. reported that ANGPTL8 increases lipolysis and regulates postprandial TG and fatty acid metabolism, owing to the assistance in activation of ANGPTL3 [9]. On the other hand, Zhang et al. showed that ANGPTL8 reduces TG clearance via LPL inhibition, thereby increasing the serum TG content [3]. In the present study, ANGPTL8 concentration were positively associated with TG levels in Korea children at baseline. Our results are consistent with previous cross-sectional data that suggest a relationship between ANGPTL8 and lipid profile, especially circulating TG concentrations [21]. Furthermore, we observed a significant association between baseline ANGPTL8 concentrations and future changes in serum TG levels in humans in the longitudinal study. Further studies are needed to explore the biological mechanisms involving ANGPTL8 in the pathogenesis of hypertriglyceridemia.

ANGPTL3 mRNA is expressed exclusively in the livers of mice and humans, and plays several roles in lipid transport and metabolism. In a mouse study, adenovirus-mediated overexpression of ANGPTL3 or treatment of recombinant ANGPTL3 significantly elevated plasma levels of nonesterified fatty acids (NEFA), total cholesterol and TG [26]. Also, ANGPTL3 increased very low density lipoprotein (VLDL)-TG levels by inhibiting LPL activity [27]. In addition, ANGPTL3 binds to adipocytes and causes the release of NEFA through activating lipolysis [28]. Moreover, ANGPTL3 acts as an inhibitor of endothelial lipase (EL) and hence decreases plasma HDL cholesterol levels [29]. Furthermore, ANGPTL3 deficiency is associated with increased insulin sensitivity and decreased serum free fatty acids, suggesting a major role of ANGPTL3 in modulating both lipid and glucose metabolism [30]. Yilmaz et al. reported that ANGPTL3 concentrations were significantly higher in patients with nonalcoholic steatohepatitis (NASH) and positively associated with HOMA-IR [31]. In a cross-sectional study including 1770 Caucasian subjects, plasma ANGPTL3 and ANGPTL4 concentrations showed different relationships with plasma lipids [32]; ANGPTL3 was positively associated with LDL cholesterol and HDL cholesterol and not with metabolic syndrome traits including TGs. Using comparative analysis of genome-wide association studies, Angelakopoulou et al. demonstrated that a single nucleotide polymorphism of ANGPTL3 (rs12042319) was associated with coronary heart disease risk (OR = 1.11; 95 % CI = 1.03 ~ 1.19), total cholesterol, LDL-cholesterol, TG, and interleukin-6 [33]. In this study, we observed that ANGPTL3 concentrations were significantly associated with fasting insulin concentrations and HOMA-IR levels, although an association with lipid profiles was not found. Previously, Robciuc et al. reported that ANGPTL3 deficiency resulted in increased insulin sensitivity, which is associated with increased lipoprotein lipase activity and low circulating free fatty acids [30] and Wang et al. showed that Angptl3−/− mice increased insulin sensitivity compared with wild-type animals by euglycemic–hyperinsulinemic clamp studies [34]. In addition, baseline ANGPTL3 levels were not related to changes of anthropometric and biochemical variables after the 3 year follow-up period in this study. In particular, there was no significant relationship between circulating ANGPTL8 and ANGPTL3 concentrations in Korean children (*r* = −0.073, *P* = 0.265).

## Limitations

First, the number of subjects might not have been sufficient to detect subtle changes of anthropometric and metabolic profiles in children. Second, the observational nature of our study did not allow direct proof of causality. Third, although the Tanner stage is accepted as the gold standard, we used self-reported Tanner staging because of the nature of the school-based examination. Third, serum betatrophin levels were determined only via EIAAB ELISA, measuring antibodies of N-terminus, without verification by another ELISA of C-terminus and western blotting. However, the present study also had several strengths, including the prospective study design and the accurate method used to measure study variables. Furthermore, research with children is often not affected by certain lifestyle factors, such as alcohol consumption and smoking, and may provide information about the early initiating stages of the development of metabolic disorders induced by risk factors.

## Conclusions

This prospective study, for the first time, showed that baseline ANGPTL8 concentrations were associated with baseline and future changes of TG levels in Korean boys and girls after a 3 year follow-up after adjustment for confounding factors. Future studies are needed to confirm our results in a larger population for a longer period and in other ethnic groups.

## Abbreviations

ANGPTL: angiopoietin-like protein
HOMA-IR: homeostasis model assessment of insulin resistance
K-MOSES: Korean Metabolic disorders and Obesity Study in Elementary School children
LPL: lipoprotein lipase
RIFL: refeeding-induced fat and liver protein
TG: triglycerides
